# Length ratios of the distal triceps tendon: an anatomical study for the assessment of tendon length in chronic defect situations

**DOI:** 10.1016/j.jseint.2025.101434

**Published:** 2025-12-20

**Authors:** Christopher Wahlers, Kai Hoffeld, Martin Scaal, Tim Leschinger, Lars Peter Müller, Michael Hackl

**Affiliations:** a University of Cologne, Faculty of Medicine, Cologne, Germany; b Department of Orthopaedic, Trauma, and Plastic Surgery, University Hospital of Cologne, Cologne, Germany; c Department of Anatomy, University Hospital of Cologne, Cologne, Germany; d Department of Orthopaedic and Trauma Surgery, University Medical Centre Mannheim, Mannheim, Germany; e Medical Faculty Mannheim, Mannheim, Germany; f University of Heidelberg, Mannheim, Germany

**Keywords:** Triceps, Tendon, Rupture, Refixation, Length, Correlation, Triceps tendon anatomy, Triceps tendon

## Abstract

**Background:**

Chronic triceps tendon ruptures are rare but challenging to treat due to limited muscle mobilization and difficulty in accurately assessing tendon length. Proper tendon length restoration is essential for maintaining physiological muscle tension, as excessive tendon length may lead to weakness and extension lag with reduced functional stability during elbow extension, whereas insufficient length can restrict elbow flexion and alter joint kinematics. This study aimed to examine the length ratios of the distal triceps tendon and aponeurosis in relation to bony landmarks to provide an intraoperative reference for tendon length assessment in chronic defect situations.

**Methods:**

A total of 54 embalmed cadaveric upper limbs (male: 54%, female: 46%) were dissected. The following anatomical parameters were measured: the length of the distal triceps tendon from the olecranon (OL) tip to the musculotendinous junction, the lengths of the medial and lateral distal triceps aponeurosis, the length of the ulna (UL) and radius (RA), and the width of the OL and the distal humerus (intercondylar width). Pearson correlation coefficients were used to analyze relationships between tendon/aponeurosis lengths and bony landmarks.

**Results:**

The mean length of the distal triceps tendon was 40 ± 15 mm, while the medial and lateral triceps aponeurosis measured 150 ± 17 mm and 115 ± 18 mm, respectively. Moderate correlations were observed between the distal triceps tendon length and both the width of the distal humerus (r = 0.42, *P* = .002) and the width of the OL (r = 0.36, *P* = .007). The lateral aponeurosis length showed strong correlations with UL length (r = 0.64, *P* < .001), RA length (r = 0.54, *P* < .001), and distal humerus width (r = 0.51, *P* < .001), while a moderate correlation was found with OL width (r = 0.41, *P* = .002). The medial aponeurosis length strongly correlated with the OL width (r = 0.54, *P* < .001) and UL length (r = 0.52, *P* < .001), and moderately with the RA length (r = 0.49, *P* < .001) and distal humerus intercondylar width (r = 0.44, *P* < .001).

**Conclusion:**

This study demonstrates that the lateral aponeurosis length correlates strongly with UL length, making it a particularly reliable reference for estimating the physiological triceps length in chronic defect situations. These findings provide an important anatomical basis for achieving precise triceps length restoration, which is essential for optimal clinical outcomes in reconstructive surgery.

Chronic triceps tendon ruptures (Fig. 1) represent a rare but extremely challenging entity to treat.^3^ This is due to delayed diagnosis, tendon retraction, and poor tissue quality, which complicate surgical repair.^7^ The systematic review by Dunn et al^5^ highlights that late diagnosis^6^ often requires more complex reconstructive techniques.^4^^,^^13^ In addition, patients with underlying risk factors such as renal disease, hyperparathyroidism,^11^^,^^16^ or anabolic steroid^10^^,^^15^ use may experience compromised healing, leading to higher rates of postoperative weakness and potential rerupture despite surgical intervention. The progressive fibrosis and shortening of the tendon over time make direct repair difficult, necessitating alternative strategies such as tendon grafting or augmentation.^4^^,^^6^^,^^7^^,^^9^^,^^13^ Therefore, accurately assessing tendon length is crucial for successful reconstruction.

**Figure 1:**
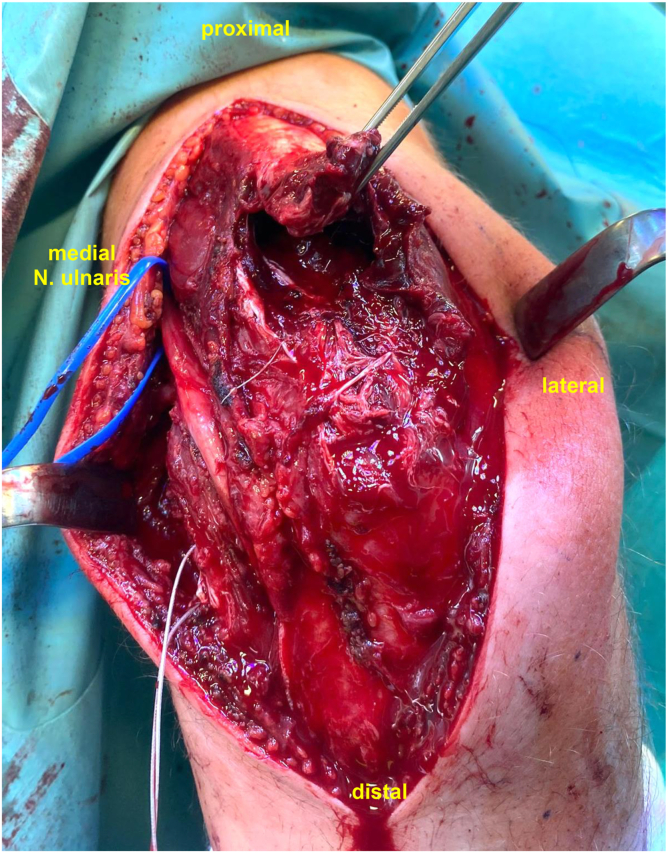
Intraoperative distal triceps tendon rupture in a chronic defect situation. Identifying the correct tendon insertion is challenging.

To make it more difficult, the triceps brachii tendon is a complex anatomic structure composed of three distinct muscle heads—long, lateral, and medial—that converge at the olecranon tip (OL). Madsen et al^8^ challenged the traditional view of a single, unified triceps tendon by demonstrating that the medial head has a separate deep insertion beneath the common tendon of the long and lateral heads. Dissection of cadaveric elbows revealed a fascial plane that distinctly separates the medial head, which remains muscular until its insertion, blending minimally with tendinous fibers. Despite this anatomical distinction, histological analysis confirmed that the tendons merge at their OL attachment. This differentiation has clinical relevance, as isolated ruptures of the deep medial head may not be apparent upon standard examination and require specific assessment techniques, particularly with the elbow in full flexion.^8^

Besides the limited ability to mobilize the muscle bellies, the assessment of correct tendon length poses a particular technical difficulty in the chronic situation, significantly influencing the physiological tension of the muscle and, consequently, the clinical outcome.

Previous anatomical studies have described the complexity and variability of the distal triceps tendon insertion. Windisch et al^14^ demonstrated that the tendon is multilayered, with overlapping contributions from the long, lateral, and medial heads, highlighting substantial interindividual variation. Barco et al^2^ further characterized the insertional footprint on the OL and emphasized its surgical relevance for repair techniques and implant placement. These findings underline the anatomical variability of the triceps insertion and the need for quantitative morphometric reference data to guide tendon length restoration.

The aim of this study was, therefore, to examine the length ratios of the distal triceps tendon and aponeurosis in relation to bony landmarks to establish an intraoperative guide for assessing the appropriate tendon length in a chronic defect situation.

## Methods

Fifty-four formalin-fixed cadaveric arms (male:female = 54%:46%) were analyzed. All specimens were embalmed using a 4% formaldehyde-based solution according to the standard institutional protocol of the Department of Anatomy, University of Cologne. Although formalin fixation reduces tissue elasticity, it preserves overall morphology and relative proportions, allowing for reliable anatomical length ratio assessment. To prevent dehydration, all specimens were kept covered with moist gauze throughout dissection and measurement. Formalin-fixed cadaveric specimens were selected to ensure structural preservation and reproducible morphometric relationships; although fixation alters elasticity, it remains appropriate for ratio-based anatomical studies.

### Skin and subcutaneous tissue were completely removed from all specimens

**Figure 2:**
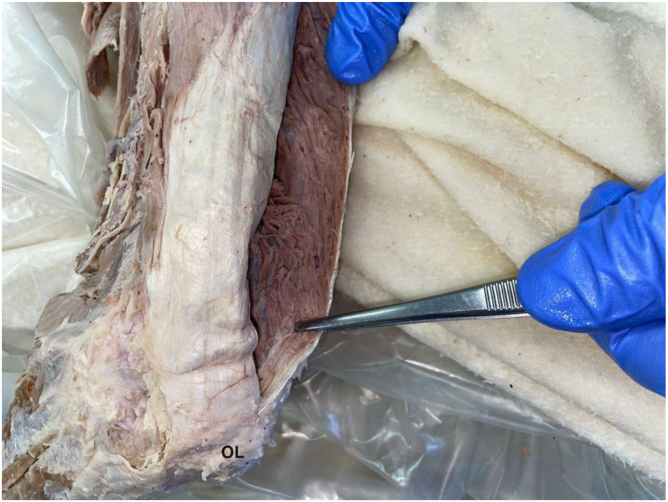
Determination of the distal triceps tendon length from the olecranon tip to the musculotendinous junction.

**Figure 3:**
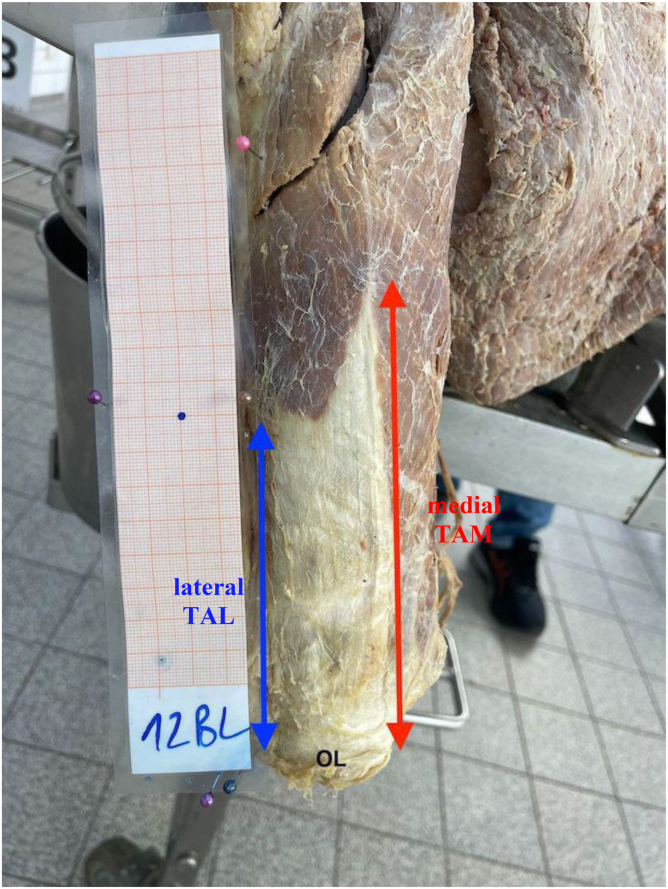
Aponeurosis of the distal triceps. Lateral aponeurosis length (TAL, *blue*) and medial aponeurosis length (TAM, *red*) measured from the OL. *OL*, olecranon tip; *TAL*, triceps aponeurosis lateral; *TAM*, triceps aponeurosis medial.

The following parameters were measured:

Tendon and aponeurosis lengths: The triceps tendon length was defined as the distance from the OL to the most proximal visible transition between tendon and muscle fibers of the common tendon formed by the long and lateral heads of the triceps brachii. The deep medial head, which inserts more distally and remains largely muscular until its OL attachment, was not included in this measurement to ensure consistency and reproducibility. The lengths of the distal triceps aponeurosis were measured medially and laterally (Figs. 2 and 3).

Bony dimensions: Length of the radius (RA) from the radial head to the radial styloid process; length of the ulna (UL) from the OL to the ulnar styloid process; width of the OL, measured at the level of its widest transverse diameter just distal to the OL, corresponding to the region of the triceps insertion footprint as described by Wegmann et al^12^; and width of the distal humerus (intercondylar width, measured as the distance between the medial and lateral epicondyles).

Measurements were conducted with the elbow positioned at 90° flexion using a predefined standardized protocol. Two independent observers, both trained orthopedic surgeons, performed all measurements. Each parameter was measured three times per observer, and the mean of these readings was used for statistical analysis. If a deviation greater than 1 mm occurred between observers, the measurement was repeated jointly to reach consensus and ensure consistency. The collected data were analyzed using Pearson correlation coefficients to assess the relationships between tendon/aponeurosis lengths and the bony reference values.

This is a basic science study. Statistical analysis was performed with IBM SPSS Statistics Version 29.0 for Mac (IBM Corp, Armonk, NY, USA). We report median (range). A value of *P* < .05 was considered to be statistically significant. Correlation was analyzed by Pearson correlation coefficient.

Ethical approval for this study was given by the Institutional Review Board of the University Cologne (ID-number: 23-1488).

## Results

The length of the distal triceps tendon was 40 ± 15 mm. The length of the distal triceps aponeurosis was 150 ± 17 mm medially and 115 ± 18 mm laterally.

Strong correlations were observed between the lateral aponeurosis length and the UL length (r = 0.64, *P* < .001), as well as the medial aponeurosis length with the OL width (r = 0.54, *P* < .001).

Moderate correlations were noted for the medial aponeurosis and RA length (r = 0.49, *P* < .001). Table I summarizes the key findings.

**Table I:** Pearson correlation coefficients between tendon/aponeurosis lengths (TTL, TAL, TAM) and bony reference measurements (ICW, RA length, UL length, and olecranon width [OL]).

	ICW	Length of RA	Length of UL	Width of the OL	TAM	TAL
TTL						
Pearson *r*	0.42	0.24	0.23	0.36	0.15	0.03
*P* value	.002	.08	.09	.007	0.28	0.82
TAL						
Pearson *r*	0.51	0.55	**0.64**	0.41		
*P* value	<.001	<.001	**<.001**	.002		
TAM						
Pearson *r*	0.44	0.49	0.52	0.54		
*P* value	<.001	<.001	<.001	<.001		

*ICW*, intercondylar width; *RA*, radius; *UL*, ulna; *OL*, olecranon tip; *TTL*, triceps tendon length; *TAL*, triceps aponeurosis lateral; *TAM*, triceps aponeurosis medial.

Values are Pearson *r* with corresponding *P* values. Bold values indicate statistical significance (*P* < .05, two-tailed).

### Key findings

• Lateral aponeurosis length: correlated strongly with UL and RA lengths, as well as intercondylar width.
• Medial aponeurosis length: correlated strongly with OL width and UL length.
• Overall tendon length: displayed moderate correlations with OL width and distal humerus width.
• The ratio of lateral aponeurosis length to UL length (0.44:1) represents an averaged proportional relationship derived from the correlation analysis rather than an absolute anatomical constant.

## Discussion

This study analyzed 54 formalin-fixed cadaveric upper limbs to identify reproducible anatomical relationships between triceps tendon and aponeurosis lengths and corresponding bony landmarks. The results demonstrated that the lateral aponeurosis length strongly correlates with UL length, providing a reliable reference for intraoperative assessment of physiological triceps tendon length in chronic defect situations. This finding offers practical relevance for reconstructive elbow surgery by facilitating the restoration of optimal muscle tension during triceps repair. Because formalin fixation alters viscoelastic properties, absolute tendon lengths must be interpreted cautiously. Nevertheless, the proportional relationships between triceps structures and osseous landmarks appear robust and clinically meaningful. The findings of this study underscore the clinical significance of bony landmarks, particularly the UL length, in reconstructing the distal triceps tendon. The strong correlation between the lateral aponeurosis length and the UL length (Pearson r = 0.64, *P* < .001) highlights its reliability as a reference parameter, with a ratio of 0.44:1. This relationship allows for an accurate estimation of the physiological triceps length in chronic defect situations, facilitating an anatomical restoration of the triceps length proportions—a critical factor in achieving optimal clinical outcomes.

### Possible clinical implications

Because of the lentiform shape of the triceps aponeurosis and the curved contour of the OL, defining precise measurement endpoints is inherently difficult. Thus, the reported 0.44:1 ratio should be regarded as an approximate proportional relationship that reflects an average correlation rather than a fixed anatomical constant.

While the present cadaveric study provides baseline anatomical reference ratios, these data do not account for pathological alterations occurring in chronic rupture situations, such as tendon crimping, fibrosis, or differential retraction of the triceps heads. In vivo, such changes may modify the original length relationships and should therefore be considered during reconstruction. The proposed ratios should thus be interpreted as anatomical orientation values that may guide, but not rigidly determine, intraoperative tendon length restoration.

The ability to use the UL as a reliable reference point simplifies the complex decision-making process during surgery. Unlike intraoperative trial-and-error estimations or reliance on contralateral measurements, using UL length as a reference provides a standardized, objective approach. While this method is promising, further research is required to determine its reliability in live tissue and dynamic settings.^8^ This could be advantageous not only for reducing intraoperative time but also for minimizing the risk of postoperative complications, such as over- or under-tensioning of the tendon and the following loss of range of motion or extension strength.

The results align with existing literature that emphasizes the importance of restoring anatomical length and tension in tendon reconstructions.^1^^,^^5^^,^^8^ Similarly, systematic reviews by Dunn et al^5^ and Alkhalfan et al^1^ have highlighted the challenges associated with diagnosing and surgically managing chronic triceps tendon ruptures, particularly regarding delayed diagnosis and optimal repair techniques. However, this study adds to the field by quantifying specific correlations between tendon/aponeurosis lengths and bony landmarks, which had not been thoroughly investigated before.

Further research should explore the integration of these findings into surgical workflows. For instance, incorporating preoperative imaging techniques to measure UL length and other bony landmarks could further enhance surgical planning and execution. In addition, biomechanical studies could evaluate how variations in reconstructed tendon length influence extension strength, range of motion, and long-term outcomes.

Exploring advanced imaging modalities, such as ultrasound or magnetic resonance imaging, could also refine the measurement of these anatomical landmarks. These tools may provide a more precise, patient-specific approach to tendon reconstruction. Finally, expanding the study to include larger and more diverse populations would help confirm the universality of these findings.

## Conclusion

The results of this study demonstrate that the length of the lateral aponeurosis of the distal triceps strongly correlates with the length of the UL (ratio 0.44:1). This finding indicates that it is particularly reliable for accurately assessing the physiological length of the triceps in chronic defect situations. Therefore, it provides a crucial anatomical basis for achieving the restoration of triceps length ratios, which is pivotal for clinical outcomes.

## Limitations

Several limitations have to be addressed. The exclusive use of formalin-fixed specimens alters tendon and aponeurosis elasticity due to tissue shrinkage and stiffness. As a result, absolute length measurements cannot be considered physiological and should be interpreted with caution. Nevertheless, the proportional relationships between tendon or aponeurosis lengths and bony reference points are likely to remain largely unaffected, supporting the validity of the observed morphometric ratios. Future studies should aim to validate these findings in a clinical setting using intraoperative measurements and functional outcome assessments. In addition, assessing variations in soft tissue elasticity and dynamic muscle behavior in living subjects would improve clinical applicability.

The study's sample size, while adequate for identifying statistically significant correlations, may not fully capture the variability seen in diverse populations. For example, anatomical differences related to age, gender, and activity level were not controlled, which could influence the generalizability of the results. Furthermore, while the strong correlation between UL length and lateral aponeurosis length suggests a promising intraoperative guide, it remains unclear how these measurements translate into improved surgical outcomes. Prospective clinical trials are essential to establish whether the use of these landmarks significantly enhances functional recovery and reduces revision rates. Despite these limitations, this study provides reproducible baseline data that may serve as a foundation for future clinical and biomechanical validation.

## Disclaimers:

Funding: No external funding or grants were received for this study. No commercial entity provided equipment or other items for the conduct of this study.

Conflicts of interest: Michael Hackl is a consultant for Arthrex, Medartis, PrehApp and Sporlastic. He also received research support from IBRA and OTC. The other authors, their immediate families, and any research foundation with which they are affiliated have not received any financial payments or other benefits from any commercial entity related to the subject of this article. None of the authors, or any members of their families, have received financial remuneration related to the subject of this article.
